# Evaluation of Soft Tissue Sarcoma Tumors Electrical Conductivity Anisotropy Using Diffusion Tensor Imaging for Numerical Modeling on Electroporation

**Published:** 2016-06-01

**Authors:** K. Ghazikhanlou-sani, S. M. P. Firoozabadi, L. Agha-ghazvini, H. Mahmoodzadeh

**Affiliations:** 1 PhD student of Medical Physics, Tarbiat Modares University, Medical Physics Department, Tehran, Iran; 2 Professor of Biomedical Engineering, Tarbiat Modares University, Medical Physics Department, Tehran, Iran; 3 Assistant Professor of Radiology, Department of Radiology, School of Medicine and Shariati Hospital, Tehran University of Medical Sciences, Tehran, Iran; 4 Assistant Professor of General Surgery, Imam Khomeini Hospital, Tehran University of Medical Sciences, Tehran, Iran

**Keywords:** Diffusion Tensor Imaging, Electrical conductivity, Anisotropy, Sarcoma tumors

## Abstract

**Introduction:**

There is many ways to assessing the electrical conductivity anisotropy of a tumor. Applying the values of tissue electrical conductivity anisotropy is crucial in numerical modeling of the electric and thermal field distribution in electroporation treatments. This study aims to calculate the tissues electrical conductivity anisotropy in patients with sarcoma tumors using diffusion tensor imaging technique.

**Materials and Method:**

A total of 3 subjects were involved in this study. All of patients had clinically apparent sarcoma tumors at the extremities. The T1, T2 and DTI images were performed using a 3-Tesla multi-coil, multi-channel MRI system. The fractional anisotropy (FA) maps were performed using the FSL (FMRI software library) software regarding the DTI images. The 3D matrix of the FA maps of each area (tumor, normal soft tissue and bone/s) was reconstructed and the anisotropy matrix was calculated regarding to the FA values.

**Result:**

The mean FA values in direction of main axis in sarcoma tumors were ranged between 0.475–0.690.  With assumption of isotropy of the electrical conductivity, the FA value of electrical conductivity at each X, Y and Z coordinate axes would be equal to 0.577. The gathered results showed that there is a mean error band of 20% in electrical conductivity, if the electrical conductivity anisotropy not concluded at the calculations. The comparison of FA values showed that there is a significant statistical difference between the mean FA value of tumor and normal soft tissues (P<0.05).

**Conclusion:**

DTI is a feasible technique for the assessment of electrical conductivity anisotropy of tissues.  It is crucial to quantify the electrical conductivity anisotropy data of tissues for numerical modeling of electroporation treatments.

## Introduction

Soft tissue sarcomas are malignant tumors of soft tissues that arise from a variety of cells of mesenchymal origin and represent less than 1% of all malignant tumors in the whole human body. The vast majority of soft tissue sarcomas arise in the extremities[1, 2]. Their natural history is partially known and clinical decisions rely on a few simple and well-recognized prognostic factors such as size, grading, and location[3]. Soft tissue sarcomas of the extremities are rare and amputation was the treatment of choice for them[4].

Electroporation involves the application of an electric field across the cell membrane to create nanoscale pores, thereby increasing membrane permeability[5]. Typically, these pores close shortly after application of the electric field; this reversible electroporation phenomenon has been widely used to facilitate gene transfer[6-8] and drug delivery[7, 8]. However, when the electric field across the cell membrane is sufficiently strong, the cell membrane pores do not reseal, leading to a loss of homeostasis and eventual cell death; this process has been described as irreversible electroporation (IRE)[9]. IRE has been applied as a tissue ablation modality and may offer multiple potential advantages compared with the other treatment modalities (such as surgery, radiation therapy).  IRE does not suffer the normal soft tissue and can lead to indistinct margins between treated and untreated tissues[9-12].

The vast majority of variables effected the field distribution on electroporation studies and the experimental testing of all variables is not practically possible. So the numerical modeling studies perform for optimization of treatments[10-12].

One of the important variables affecting the results of electroporation is the electrical conductivity anisotropy. There is many ways to assessing the electrical conductivity anisotropy of the tumor (such as impedance tomography, direct electrical measurement of electrical conductivity using electrodes, etc). Invasiveness of the direct electrical measurement and low special resolution of the impedance tomography techniques are the main restrictions of the mentioned techniques[13, 14].

Diffusion Tensor Imaging (DTI) is a useful technique for the noninvasive structural characterization of various anisotropic tissues[14]. This method involves estimation of the diffusion tensor using a series of diffusion-weighted imaging (DWI) images. Once the tensor is estimated, the eigenvalues and eigenvectors provide information concerning effective diffusivity along three orthogonal directions[15, 16].

The diffusion coefficient measured in magnetic resonance is the so-called “apparent diffusion coefficient” or ADC[15]. The word “apparent” is added because other factors than random diffusion may influence the mobility of water. The ADC is an average of the water mobility in all directions (if the experiment takes all 3 spatial axes into account)[16].

The fractional anisotropy (FA) is a measure of the degree of directionality of diffusion and could be calculated using the ADC maps. Its values range from 0 (no directional dependence of the diffusion) to 1 (diffusion along a single direction). In the other expression FA reflects the degree of anisotropic diffusion[15, 16].

In most electroporation numerical modeling studies, the electrical conductivity anisotropy is not considered. However, applying the values of tissue electrical conductivity anisotropy is crucial in numerical modeling of electroporation treatments. 

This study aims to calculate the conductivity anisotropy of the soft tissue, bone and tumors in patients with sarcoma tumors using FA maps derived from DTI images. This conductivity anisotropy could be used at numerical modeling of electroporation treatments (especially for treatments of sarcoma tumors). 

## Material and Methods

A total of 3 subjects were involved in this study. All of patients had clinically apparent sarcoma tumors at the extremities. The inclusion criteria were the presence of a sarcoma tumor/s at the extremities (dimensions lesser than 5 cm convenient for electroporation treatments) and the exclusion criteria were standard exclusion criteria for MRI including presence of metalwork or pacemaker, inability of patients to establish the state of immobility. The subject’s was positioned in the coil in such a way that the tumor area of the extremities was in the center of the coil. The location of the tumor was visible and was indicated by the consultant physician. 

The study was approved by Tarbiat Modares Research Ethics Board in accordance with the Declaration of Helsinki. 

MRI was performed at 3-Tesla (T) (Siemens Medical, Siemens Healthcare, Erlangen, Germany) using a multi-coil with multi-channel system (existing at Imam Khomeini Hospital, Tehran, Iran). Patients underwent T1-weighted, T2-weighted sequences of the tumour area in axial, coronal and sagittal planes prior to DTI. The acquisition time for each DTI measurement was approximately five minutes. 

The MRI parameters of each imaging technique were indicated at Table 1.

**Table 1:** The imaging parameters of T1, T2 and DTI techniques.

	**Mprage-T1**	**T2**	**DTI**
**Slice thickness**	0.9 mm	5 mm	4 mm
**TR**	2300 ms	3800 ms	5000 ms
**TE**	4.76 ms	92 ms	90 ms
**TI**	1100 ms	-------	--------
**Phase resolution**	100%	70%	100%
**Base resolution**	256	320	80
**Band width**	130 Hz/Px	252 Hz/Px	1524 Hz/Px
**Echo spacing**	10.8 ms	10.2 ms	--------
**FOV read**	240 mm	240 mm	240 mm
**b-values**	---------	----------	0, 800 and 1000 s/mm^2^

**Figure 1:**
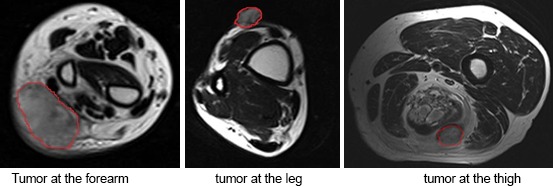
Tumor area of different patients

The tumor area of different patients on T2 images has been shown in Figure 1.

**Figure 2:**
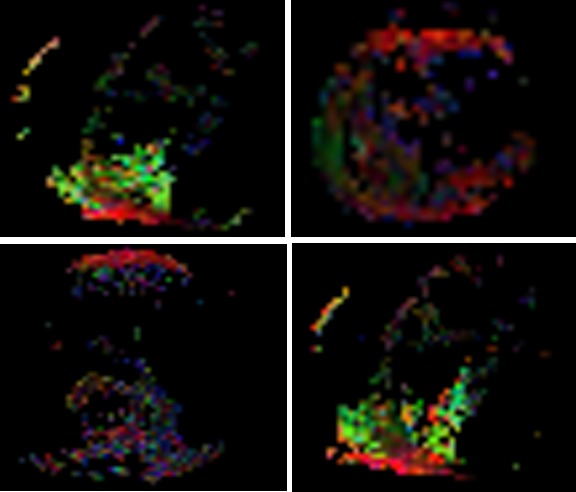
The selected FA maps of study participates patients

The FA maps were derived from ACD maps of DTI images using the FSL (FMRI software library) software that was implemented on the above mentioned MRI system. Some examples of FA maps are shown in Figure 2. At these images, the direction of the fractional anisotropy is given by color coding, red is left to right, green is front to back, and blue is head to foot. The value of FA (ranged between 0 and 1) at each pixel area was determined using the FSL software.

**Figure 3:**
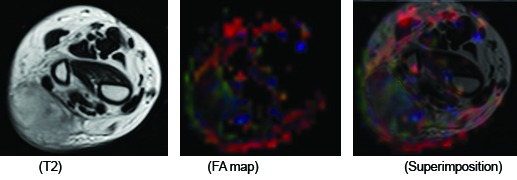
The selected FA maps of study participates patients

At the next stage for determination of tumor, bone and soft tissue borders on FA maps, the MIPAV (Medical Image Processing, Analysis, and Visualization) application were used. The MIPAV application enables quantitative analysis and visualization of medical images of numerous modalities such as PET, MRI, CT, or microscopy. Using MIPAV the T1, T2 and FA maps could be matched on together and the border of the tumor, soft tissue and bone/s could be identified on FA maps. The matching method of the DTI and T2 images using the MIPAV software are shown in Figure 3.

With this method, the area of the tumor, bones and the normal soft tissue in all the pictures were removed from the map and rendered to the MATLAB software. Then the 3D matrix of the FA maps of each area (tumor, normal soft tissue and bone/s) was reconstructed. Then the mean anisotropy matrix was calculated regarding to the FA values. By averaging the FA values at each direction the mean value of FA was calculated at three axes. With this method, the amount of anisotropy is obtained in the X, Y and Z axes that are the same axes of imaging coordinate. 

If the electric field was not applied in the same directions of main axes of imaging coordinate, the matrix of anisotropy will change and with rotation of the coordinate axes, each of main components (for example X) will have new components in new coordinate (Xx’, Xy’ and Xz’).

A vector in terms of the Cartesian unit vectors (ijk) can be written as:

A=iAx+jAy+kAz

iAx, jAy and kAz are the mean value of FA values along the X (left to right), Y (front to back) and Z (head to foot) axis.

A vector could be written in terms of the unique vectors (i’ j’ k’) as follows:

A=i’ Ax’+j’ Ay’+k’ Az’

Ax’=A.i’=(i.i’ )Ax+(j.i’ )Ay+(k.i’ )Az

Ay’=A.j’=(i.j’ )Ax+(j.j’ )Ay+(k.j’ )Az

Az’=A.k’=(i.k’ )Ax+(j.k’ )Ay+(k.k’ )Az

Ax=A.i=(i’.i)Ax’+(j’.i)Ay’+(k’.i)Az’

Ay=A.j=(i’.j)Ax’+(j’.j)Ay’+(k’.j)Az’

Az=A.k=(i’.k)Ax’+(j’.k)Ay’+(k’.k)Az’

The transformation matrix from the old coordinate system to the new coordinate system could be written as follows:

$$\begin{array}{c} A_{x´}\\ A_{y´}\\ A_{z´} \end{array}=\left[\begin{array}{ccc} i.i´ & j.i´ & k.i´\\ i.j´ & j.j´ & k.j´\\ i.k´ & j.k´ & k.k´ \end{array}\right]\left[\begin{array}{c} A_{x}\\ A_{y}\\ A_{z} \end{array}\right]$$

The new coordinate system is matched on the old coordinate system, but there is rotation of X, Y and Z axes.

Now, let’s imagine that the z-axis of coordinate system rotate φ ° counterclockwise (CCW).

$$i.i^{'}=j.j^{'}=cos\emptyset$$

$$j.j^{'}=i.i^{'}=sin\emptyset$$

k.k’=0

The inner product of “k” in “I” and “j” is zero. So the transformation matrix around z axis about φ° is: 

$$\left[\begin{array}{ccc} \cos\varphi & \sin\varphi & 0\\ -\sin\varphi & \cos\varphi & 0\\ 0 & 0 & 1 \end{array}\right]$$

If XZ axis rotates around Y-axis by θ°, as the same reasoning:

$$\left[\begin{array}{ccc} \cos\theta & 0 & -\sin\theta \\ 0 & 1 & 0\\ \sin\theta & 0 & \cos\theta \end{array}\right]$$

If YZ axis rotates around X-axis by α °, will be:

$$\left[\begin{array}{ccc} 1 & 0 & 0\\ 0 & \cos\alpha & \sin\alpha \\ 0 & -\sin\alpha & \cos\alpha \end{array}\right]$$

By multiply these above mentioned three matrixes, the final transformation matrix is obtained as follows: 

O=cos⁡θcos⁡φcos⁡θsin⁡φcos⁡α cos⁡θsin⁡φsin⁡α-sin⁡θcos⁡α-sin⁡φcos⁡φcos⁡αcos⁡φsin⁡αsin⁡θcos⁡φ  sin⁡θcos⁡φcos⁡α-sin⁡αcos⁡θ    sin⁡θcos⁡φsin⁡α+cos⁡θcos⁡α

And:

$$\left[\begin{array}{c} A_{x´}\\ A_{y´}\\ A_{z´} \end{array}\right]=\;o\left[\begin{array}{c} A_{x}\\ A_{y}\\ A_{z} \end{array}\right]\;$$

**Figure 4:**
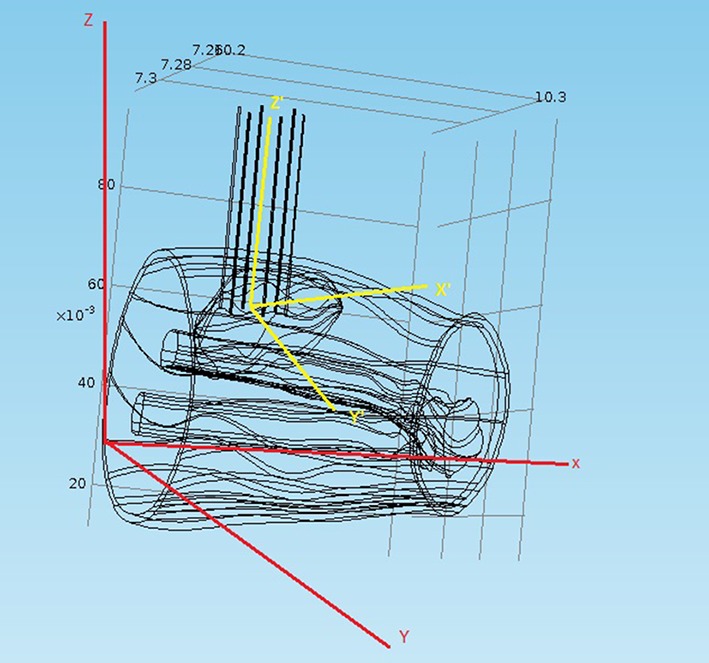
Determination of ɑ, ɵ and ɸ angles for calculation of anisotropy matrix

The value of φ, θ and α is determined regarding the application of electric current and the placement of the electrodes in the tissue. For example if an electric current is applied in the direction of the MR imaging the value of φ, θ and α would be equal to zero. So, the rotation size of the coordinate axes should be based on the angle of main axes of applied electrical current in comparison to the main axes of the MRI imaging matrix (as shown in Figure 4).

Anisotropy matrix values of electrical conductivity could be achieved by multiplying the actual amount of FA on the value electrical conductivity. 

## Results

At most Numerical modeling and simulations the electrical conductivity of the tissues are considered as isotropic. Therefore in a 3*3 matrix, only the amount of XX, YY and ZZ axes is determined as the amounts of listed below and the electrical conductivity at the other directions assumed as zero. With assumption of isotropy of the electrical conductivity, the FA value of electrical conductivity at each X, Y and Z coordinate axes would be equal to 0.577. 

After calculating the anisotropy values for different parts of the tissues (tumor, soft tissue, and bone/s) respect to the FA maps reconstructed by the DTI images, the mean FA values for each patient are summarized in Table 2.

**Table 2:** The mean value of FA at different parts of the tissues and the error rate of calculations by neglecting the anisotropy

	**Anisotropy matrix of FA maps and the error rate of calculations by neglecting the anisotropy**					
	**Soft-tissue**	**Error rate**	**Tumor**	**Error rate**	**Bone**	**Error rate**
Patient 1 (tumor at the forearm)	$\left[\begin{array}{c} \text{0.642}\\ \text{0.569}\\ \text{0.513} \end{array}\right]$	$\left[\begin{array}{c} \%11\\ \%11\\ \%1 \end{array}\right]$	$\left[\begin{array}{c} 0.690\\ 0.475\\ 0.564 \end{array}\right]$	$\left[\begin{array}{c} \%19\\ \%17\\ \%5 \end{array}\right]$	$\left[\begin{array}{c} 0.527\\ 0.507\\ 0.682 \end{array}\right]$	$\left[\begin{array}{c} \%18\\ \%12\\ \%18 \end{array}\right]$
Patient 2 (tumor at the leg)	$\left[\begin{array}{c} 0.511\\ 0.498\\ 0.70 \end{array}\right]$	$\left[\begin{array}{c} \%11\\ \%13\\ \%21 \end{array}\right]$	$\left[\begin{array}{c} 0.670\\ 0.521\\ 0.529 \end{array}\right]$	$\left[\begin{array}{c} \%16\\ \%9\\ \%8 \end{array}\right]$	$\left[\begin{array}{c} 0.492\\ 0.598\\ 0.632 \end{array}\right]$	$\left[\begin{array}{c} \%14\\ \%3\\ \%10 \end{array}\right]$
Patient 3 (tumor at the thigh)	$\left[\begin{array}{c} 0.615\\ 0.568\\ 0.547 \end{array}\right]$	$\left[\begin{array}{c} \%7\\ \%5\\ \%2 \end{array}\right]$	$\left[\begin{array}{c} 0.631\\ 0.478\\ 0.611 \end{array}\right]$	$\left[\begin{array}{c} \%9\\ \%6\\ \%17 \end{array}\right]$	$\left[\begin{array}{c} 0.651\\ 0.499\\ 0.572 \end{array}\right]$	$\left[\begin{array}{c} \%13\\ \%14\\ \%1 \end{array}\right]$

As shown in Table 2, the FA value of sarcoma tumors ranged between 0.475 and 0.690 and there is an error band of 20% in electrical conductivity (and subsequently for electric and thermal field distribution) if the electrical conductivity anisotropy not concluded at the calculations.

The comparison of FA values using ANOVA  statistical test showed that there is a significant difference between the mean FA value of tumor, normal soft tissue and bone/s (P<0.05). 

After implementation of the electrodes at the tumor in IRE numerical modeling studies and drawing the applied electric field axes (X’, Y’ and Z’), the rotation of new coordinate system relative to the initial imaging coordinate system are shown in Table 3.

**Table 3:** The rotations of applied electric field coordinate axes relative to the initial imaging coordinate axes

	**α(°)ccw**	**θ(°)ccw**	**φ(°)ccw**
Patient 1 (tumor at the forearm)	-4	36	-26
Patient 2 (tumor at the leg)	-12	10	46
Patient 3 (tumor at the thigh)	31	-18	-22

Because the main axes of the applied electrical current are not parallel to the main axes of the MR imaging technique and there are some rotations in the X, Y and Z axes (as shown in Figure 4). So, the fractional anisotropy observed in any direction (for example along the X axis) provides other components in the main axes of the new coordinate (that are defined by XX’, XY’ and XZ’ in the new coordinate).

Calculating the amount of rotation of the coordinate axes based on the above mentioned formulas, the overall anisotropy matrix for each parts of the tissues (soft tissue, tumor and bone/s) converted to the amounts of listed in Table 4.

**Table 4:** Anisotropy matrix of the tissues by applying rotation of the coordinate axes in terms of 3 by 3 matrixes

**patient**	**Anisotropy matrix of FA maps**		
	**Soft-tissue**	**Tumor**	**Bone/s**
Patient 1 (tumor at the forearm)	$\left[\begin{array}{ccc} 0.439 & 0.468 & 0.020\\ 0.403 & 0.372 & 0.152\\ 0.089 & 0.105 & 0.494 \end{array}\right]$	$\left[\begin{array}{ccc} 0.472 & 0.502 & 0.021\\ 0.337 & 0.310 & 0.127\\ 0.098 & 0.116 & 0.543 \end{array}\right]$	$\left[\begin{array}{ccc} 0.361 & 0.384 & 0.017\\ 0.259 & 0.331 & 0.136\\ 0.118 & 0.140 & 0.657 \end{array}\right]$
Patient 2 (tumor at the leg)	$\left[\begin{array}{ccc} 0.350 & 0.372 & 0.016\\ 0.353 & 0.325 & 0.133\\ 0.121 & 0.143 & 0.674 \end{array}\right]$	$\left[\begin{array}{ccc} 0.459 & 0.488 & 0.021\\ 0.370 & 0.341 & 0.139\\ 0.092 & 0.108 & 0.510 \end{array}\right]$	$\left[\begin{array}{ccc} 0.337 & 0.359 & 0.015\\ 0.423 & 0.391 & 0.160\\ 0.110 & 0.130 & 0.609 \end{array}\right]$
Patient 3 (tumor at the thigh)	$\left[\begin{array}{ccc} 0.542 & 0.107 & 0.270\\ 0.202 & 0.485 & 0.215\\ 0.169 & 0.268 & 0.446 \end{array}\right]$	$\left[\begin{array}{ccc} 0.556 & 0.110 & 0.277\\ 0.170 & 0.408 & 0.181\\ 0.189 & 0.300 & 0.498 \end{array}\right]$	$\left[\begin{array}{ccc} 0.574 & 0.113 & 0.286\\ 0.178 & 0.426 & 0.189\\ 0.177 & 0.280 & 0.466 \end{array}\right]$

By multiplying the values of the mean fractional anisotropy and electrical conductivity values for each tissue, the electrical conductivity of the tissues at each direction of a 3*3 matrix could be calculated and considered in numerical modeling. 

## Discussion

Magnetic resonance methods for measuring molecular diffusion were already developed in the 1960s and in the last decade has received increased attention in biomedical studies[16-18]. The fractional anisotropy (FA), the relative anisotropy (RA), and the apparent diffusion coefficient (ADC) each provide information describing water diffusivity. FA is the fraction of diffusivity that can be ascribed to anisotropic diffusion[17].  The FA provides information about the eccentricity of the diffusion Ellipsoid.  In general, FA scales from 0 to 1, where 0 represents the perfect sphere and 1 represents an infinitely long cylinder[17, 18].

Until now, there has been no standard DTI analysis. Most current studies use region of interest (ROI) approaches for FA quantification, which often include manual ROI delineation in combination with co-registered anatomic MR imaging series. These procedures are vulnerable to mismatch problems, partial volume errors, and inter observer differences. The clinical relevance of quantifying the precision of DTI measures has been emphasized by several authors[15, 16, 18].  To our knowledge, the reliability of the current methods of FA quantification has not yet been described.

In recent years, many studies on the relationship between the values obtained from DTI images with texture characteristics in different organs, especially the tumors are made. Takaaki et al[17] described the FA values of glioblastoma tumors, Schnapauff et al[16] evaluated the FA of soft tissue sarcomas, Zaraiskaya et al[18] utilized the DTI images for musculoskeletal injury, and very different studies evaluated the tumor response at different areas.

The results of present study showed that the FA value of sarcoma tumors ranged between 0.475 and 0.690 and there is an error band of 20% in electrical conductivity if the electrical conductivity anisotropy not concluded at the calculations.

Bhandari et al[15] at 2012 in agreement with our results demonstrated that sarcoma tumors show anisotropic characteristics and the FA of tumor differed from normal tissues. They demonstrated that the anisotropy in the direction of the muscle fibers is different from other directions. Also, at Kermarrec et al study[19] similar results were obtained.

As our results, there was a significant difference between the FA values in different patients. Schimrigk et al[20] in agreement of our findings resulted that there is significant differences between the absolute FA values in a group of patients.

Bhandari et al[15] also explain that the FA value did not differ for hypo, iso or hyperintense tumors indicating this reflects a different aspect of tumor biology which may complement other MRI parameters. We did not consider the density of the tumors at our research. They also showed that the majority of tumors showed shrinkage at one year. ADC increased indicating a reduction in cellularity mirrored by a decrease in T2 signal intensity in some lesions, though this was not statistically significant.

Schnapauff et al[16] had presented the first strong evidence of DWI also correlated with tumor cellularity extracerebrally in soft-tissue sarcomas. They also explained that DWI represents a powerful tool to provide unique information related to tumor cellularity of soft-tissue sarcomas.

Zaraiskaya et al[18] also demonstrated that there are Statistical differences for mean values of FA between controls and patients with musculoskeletal injury. They also emphasis that the mean value of FA could be calculated for characterization the degree of muscle injury.

Uhl et al[21], Chen et al[22] and Theilmann et al[23] also showed the similar results for osteosarcomas, hepatocellular carcinoma and  breast cancer, respectively.

In a larger scale study, by Dudeck et al[24], soft-tissue sarcomas monitored with DWI, a close correlation of water diffusivity and response to anticancer therapy was observed.

This preliminary data supports the hypothesis that assessment of water diffusion may potentially provide additional clinically relevant information, but requires further study. Diffusion tensor imaging offers huge potential in oncology. It is relatively easy to implement as part of a standard MR examination. Diffusion imaging is performed with fast sequences, does not require contrast media injection, and thus can be repeated frequently during therapy. 

## Conclusion

In summary we have shown that DTI is feasible for the assessment of electrical conductivity anisotropy of tissues. The error rate in estimation of electrical conductivity in different directions could increase up to 20% if the electrical conductivity anisotropy not be included in the study. Therefore, we think it is crucial to quantify the anisotropy data of tissues in numerical modeling of electroporation treatments. Using the DTI technique the anisotropy of tissues could be calculated noninvasively. Extensive recommendations towards the use of DTI in the clinic recently have been described by other studies. 
